# Effects of Pretreatment Methods of Wheat Straw on Adsorption of Cd(II) from Waterlogged Paddy Soil

**DOI:** 10.3390/ijerph16020205

**Published:** 2019-01-12

**Authors:** Mengjie Wu, Hongyu Liu, Chunping Yang

**Affiliations:** 1 School of Environmental Science and Engineering, Guangdong University of Petrochemical Technology, Maoming 525000, Guangdong, China; wumengjie@hnu.edu.cn; 2 College of Environmental Science and Engineering, Hunan University, and Key Laboratory of Environmental Biology and Pollution Control (Hunan University), Ministry of Education, Changsha 410082, Hunan, China; hyliu@hnu.edu.cn; 3 Guangdong Provincial Key Laboratory of Petrochemical Pollution Process and Control, Guangdong University of Petrochemical Technology, Maoming 525000, Guangdong, China; 4 Hunan Province Environmental Protection Engineering Center for Organic Pollution Control of Urban Water and Wastewater, Changsha 410001, Hunan, China

**Keywords:** wheat straw, pretreatment, adsorption, Cd(II) removal, soil remediation, wastewater treatment

## Abstract

Two types of pretreatment categories, namely microwave-assisted alkalization and microwave-assisted acid oxidation, were used to synthesize novel wheat straw adsorbents for the effective removal of Cd(II) in simulated waterlogged paddy soil. A systematic adsorption behavior study, including adsorption kinetics and adsorption isotherms was conducted. Results showed that wheat straw pretreated by microwave-assisted soaking of NaOH and ethanol solution obtained the highest Cd(II) removal efficiency of 96.4% at a reaction temperature of 25 ℃, pH of 7.0, initial Cd(II) concentration of 50 mg/L, and adsorbent/adsorbate ratio of 10 g/L. Sequential extraction experiment was carried out to analyze the changes of different of Cd(II) in soil, the aim of which was to study the mobility of Cd(II) and then evaluate the toxicity that Cd(II) might bring to plants. A 60-day incubation was performed to investigate the dynamic variations of soil pH and dissolved organic carbon content over incubation time. Characterization analyses revealed the morphological changes of wheat straw adsorbents, which suggested that those pretreatment methods were of significance. This study provided an environmentally friendly way to reuse agricultural wastes and remedy Cd(II) contaminated soil.

## 1. Introduction

Cadmium as one of the common hazardous heavy metals can cause potential threat to human life. It can be introduced to the environment by various anthropogenic activities, especially by industrial processes, such as mining, fossil fuel combustion, metal smelting, processing and fabricating, use of cadmium in chemical fertilizers, application of cadmium in pigments and stabilizers for plastics, and so forth [1,2]. Besides, smoking may also significantly increase human’s exposure to cadmium [3]. Therefore, it is easy for cadmium to release to the environment, especially to the aquatic environment, and, as a consequence, it will also cause damage to soil and air and become a long-term risk to ecosystems and humans [4].

Although, to some extent, the total concentration of cadmium in soil is concerned with its phyto-mammalian toxicity, the behavior, including its mobility and bioavailability, in soil plays a vital part of its threat to the environment [5]. Researches show that the uptake of heavy metals from soil is closely linked to the fractionations of those metals [6]. The toxicity of heavy metals often best associates with the activity of free metal ions. The bioavailability of heavy metals in soil is usually evaluated by their water-soluble concentration; however, the total dissolved metal concentration does not necessarily relate to the amount that is available to biota [7]. Therefore, sequential extraction procedures are applied to predict the availability of heavy metals to plants [8].

Removing heavy metal ions from aqueous solutions is a challenge for researchers. Conventional technologies that are usually used to extract heavy metal ions from wastewater include physical, chemical, and biological methods [9,10,11]. However, some of the conventional technologies have disadvantages, such as low efficiency, high cost, and secondary pollution. Thus, adsorption technology that possesses the advantages of high efficiency, low cost, and easy operation has been widely applied [12,13].

Crop stalks as an important by-product during the crops growing process have attracted a lot of attentions, not only due to their vast source, but also their easy availability, not to mention their low cost [14,15]. As Tye et al. [16] suggested in their paper, there were about 1580 million tons of crop straws production in 2016, mainly from Europe (barley and oat), the United States (corn and sorghum), and China (rice and wheat). Rich in nitrogen, phosphorus, potassium, calcium, and various organic matters, crop stalks can be used as valuable biomass resources. Moreover, crude fibers are composed of cellulose, lignin, and hemicelluloses, which are the major constitutes of plant cell walls. Because of the large quantity of active functional groups (hydroxyl, carboxyl, carbonyl, etc.) on the surface of cellulose, lignin and hemicelluloses, chelating, ion exchange, and other reactions may occur when they come into contact with heavy metal ions, like Cd(II), and by this way those heavy metal toxins may be extracted from the contaminated soils [17]. Owing to these properties, crop stalks have been used as biological adsorbents for a long time.

Previous studies have reported that, crop stalks, when pretreated by different methods, their adsorption capacity on heavy metal ions might be enhanced a lot [18,19]. For instance, Garcia et al. [20] showed that using dilute alkali could damage the structure of cellulose, hemicelluloses, and lignin in wheat straw (WS). Huijgen et al. [21] used dilute acid to pretreat WS and found that it could absorb more Cd(II) from aqueous solutions, which may due to the reason that hemicelluloses could be converted into the corresponding monosaccharide by dilute acid pretreatment. Furthermore, using microwave irradiation to pretreat WS is also a common way to achieve high adsorption capacity of Pb(II) and Cd(II) [22,23]. Those pretreatment methods could modify the surface characteristics/groups of the straws, either by removing or masking the groups or by exposing more metal-binding sites [24].

This study aimed to examine the effects of different pretreatment methods on WS adsorbents properties. Four kinds of WS biosorbents were synthesized by microwave-assisted alkalization or microwave-assisted acid oxidation. The characterization of the four biosorbents was analyzed by scanning electron microscope, Fourier Transform Infrared Spectroscopy, and Brunauer-Emmett-Teller specific surface areas measurement. Influences of Cd(II) solution pH, initial concentration, contact time, and solid/liquid ratio were investigated. Besides, adsorption kinetics, thermodynamics, and equilibrium of the biosorbents were determined.

## 2. Materials and Methods

### 2.1. Materials

Soil samples used in the study were collected from a local paddy field (28°31′2′′ N, 113°5′49′′ E) in Changsha (Hunan province, China). The soil sample was taken from the subsoil in the layer between 20 cm and 40 cm below the surface, the five-point sampling method was carried out when sampling. Stones, sticks, and big roots (diameter > 1 cm) were manually removed. After drying the soil in the air, weighed 1000 g dried soil from each sampling point, mixed, ground, and passed through a 60-mesh nylon sieve and stored in wide-mouth glass bottles. Raw wheat straw (RWS) was purchased from Zhengzhou (Henan province, China). After cutting the straws into 1–3 cm, they were washed with ultrapure water, dried at 80 °C for 24 h, ground and passed through a 35 mesh sieve, and stored in plastic hermetic bags for further use.

Cadmium sulfate (CdSO_4_·8/3H_2_O), ethanol (C_2_H_6_O), sodium hydroxide (NaOH), potassium hydroxide (KOH), sulfuric acid (H_2_SO_4_), citric acid (C_6_H_8_O_7_), acetic acid (HAc, CH_3_COOH, ≥99.5%), sodium acetate (NaAc, CH_3_COONa·3H_2_O), calcium chloride (CaCl_2_), hydrofluoric acid (HF), perchloric acid (HClO_4_), nitric acid (HNO_3_), cadmium nitrate (Cd(NO_3_)_2_·4H_2_O), and sodium nitrate (NaNO_3_) were purchased from Sinopharm Chemical Reagent Co., Ltd. and were all analytically pure grade. Ultrapure water was used in all the experiments and all of the containers were soaked in dilute nitric acid overnight and thoroughly washed with ultrapure water before using 0.1 mol/L CH_3_COOH was prepared by diluting 5.7 mL of glacial acetic acid into 94.3 mL of ultrapure water; 1 mol/L CH_3_COONa was prepared by adding 13.6 g CH_3_COONa·3H_2_O into 100 mL ultrapure water; 0.1 mol/L CaCl_2_ was prepared by adding 11.1 g anhydrous calcium chloride into 1000 mL ultrapure water; and, 100 mg/L Cd(NO_3_)_2_ was prepared by dissolving 130.5085 mg Cd(NO_3_)_2_·4H_2_O into 1000 mL ultrapure water. All of the experiments were performed in triplicate. 

### 2.2. Soil Contamination and Wheat Straw Pretreatment

CdSO_4_ was used to contaminate the soil sample so as to simulate Cd(II)-contaminated soil. Sprayed 10 mL 10% of CdSO_4_ solution several times on 100 g of above-mentioned soil to make sure the contamination was even, mixed the soil thoroughly, and air dried it at room temperature. The simulated Cd(II)-contaminated soil was taken for use after 30 days to balance the concentration of Cd(II) between solid and liquid phase in soil. The Cd(II) concentration of the contaminated soil was 9.47 mg/kg.

Four kinds of pretreatment methods were applied to modify the WS, and those methods might fall into two major categories: microwave-assisted alkali saponification and microwave-assisted acid oxidation. Detailed steps are described, as follows. 10.0 g of WS was taken, put it into a beaker, added 100 mL of ethanol, and stirred for 24 h at 25 °C and 200 rpm. After that, we used ethanol to wash the WS three times, and then used ultrapure water to wash until the filtrate was clean. The filter residue was oven-dried at 60 °C for 24 h and the pretreated WS was labeled as EWS. Took 10.0 g of EWS, mixed with 200 mL 0.1 mol/L NaOH, stirred at room temperature for 3 h, put it into a microwave oven (P70D20TL-D4, Shunde, Guangdong, China) for 5 min, and then used ultrapure water to wash the filter residue until its pH was neutral. Oven-dried at 60 °C for 24 h and labeled as ENMWS. The adsorbent EKMWS was synthesized by substituting 0.1 mol/L KOH for 0.1 mol/L NaOH. As for the pretreatment methods of microwave-assisted acid oxidation, 10.0 g of EWS was taken, mixed with 100 mL 0.5 mol/L H_2_SO_4_, kept at 70 °C, stirred for 2 h, microwaved for 5 min, and then used ultrapure water to wash the filter residue until its pH was neutral. It was iven-dried at 60 °C for 24 h and then labeled as EHMWS. Citric acid was used to replace H_2_SO_4_ so as to prepare adsorbent ECMWS. The raw WS was used as the control group.

### 2.3. Incubation Experiment

The incubation experiment was carried out by weighing 100.0 g of Cd(II)-contaminated soil to each 500 mL beaker, adding five kinds of pretreated WS with a straw-soil ratio of 1:50, respectively, mixing them evenly, and adding water to submerge the soil (150% of the soil moisture content). To make an anoxic environment, plastic wrap was used to seal the beakers. All of the samples added with five types of WS were placed into a constant temperature incubator of 25 °C and the incubation time was set as 60 days. Approximately 20.0 g of wet soil (to obtain about 10.0 g dry soil) was taken out from each beaker on the 3rd, 6th, 10th, 20th, 40th, and 60th day of the incubation (when using the HF-HCl-HClO_4_-HNO_3_ digestion method to measure the residue Cd(II) in the mixture of soil and straw, the amount of Cd(II) adsorbed by the wheat straw would also be accounted for the residue Cd(II)). The wet soil was air-dried and ground, passed through a 35-mesh sieve, prepared for physicochemical analyses, which include pH and dissolved organic carbon (DOC), and Cd(II) content measurement. Soil pH was determined by the following operations: took 1.0 g soil to a beaker and added 5 mL of ultrapure water into it, mixed them on a magnetic stirrer for 1 min, let it sit for 30 min, and then use PHS-3C pH meter (Shanghai INESA Scientific Instrument Co. Ltd., Shanghai, China) to measure.

### 2.4. DOC Measurement and Sequential Extraction Analysis

The determination of DOC was performed according to a standard method that was provided by the environmental protection standard of the People’s Republic of China (HJ 695-2014), and the detailed operation steps were: Took 1.0 g dried and ground soil from the previous step to 50 mL polypropylene centrifuge tubes, added 20 mL of ultrapure water, and shook in a linear shaking bath with 200 rpm at 25 °C for 16 h. After that, centrifuged them at 4500 rpm for 30 min, filtered the supernatants by 0.45 μm millipore filter, and then stored at 4 °C prior to analysis. Total organic carbon analyzer (TOC-VCPH, Shimadzu Corporation, Kyoto, Japan) was used to measure the DOC content of the samples, as mentioned before.

Given that traditional sequential extraction procedures, such as Tessier and BCR sequential extraction procedure, have some flaws, to propose an optimized method seems necessary. When considering that the pH of the soil sample was around 5–6, the Toxicity Characteristic Leaching Procedure (USEPA) was referred in the experiments. Specific steps were as follows: (1) HAc-Cd: adding 20 mL of 0.1 mol/L HAc (pH = 2.88 ± 0.05) to 1.000 g sample soil, shaking them at a rotate speed of 250 rpm for 16 h at 25 °C; (2) NaAc-Cd: 10 mL of 1 mol/L NaAc (pH = 5.0) was added into the residue obtained from previous step and then shook with 250 rpm for 5 h at 25 °C; (3) CaCl_2_-Cd: adding 10 mL of 0.1 mol/L CaCl_2_ to the residue of the previous step, agitating with 250 rpm for 12 h at 25 °C; and, (4) Res-Cd: HF-HCl-HClO_4_-HNO_3_ digestion method was applied to measure the remnant content of Cd(II) in the soils. It was assumed that HAc-Cd represented acid dissolvable Cd(II), NaAc-Cd was similar to carbonate bound speciation of Cd(II), and CaCl_2_-Cd could be regarded as a combination of water-soluble and part of exchangeable fraction of Cd(II). When each step was finished, the samples were centrifuged at 3000 rpm for 15 min, the supernatants weree filtered with 0.45 μm millipore filter, and the sample solutions were stored at 4 °C for future measuring of DOC content. The residue of each step was washed by ultrapure water for three times, centrifuged, and discarded the supernatants before applying to the next step.

### 2.5. Adsorption Experiment

Adsorption kinetic experiment was conducted by mixing 1.0 g dried RWS, ENMWS, EKMWS, EHMWS, and ECMWS with 100 mL of 5, 50, 100 mg/L Cd(NO_3_)_2_ solution, respectively, which was prepared by background electrolyte solution (0.01 mol/L NaNO_3_). Shaking the mixture in a water baths shaker at 150 rpm and 25 °C, ending the shaking after 5, 15, 30, 45, 90, 240 min, and centrifuging for 10 min at 4000 rpm, and then filtering the supernatants with 0.45 μm millipore filter for further measuring.

Adsorption isotherm was determined by the following steps: weighing 1.0 g dried adsorbent, mixing it with 100 mL of 5, 20, 50, 100, 150, 200 mg/L Cd(NO_3_)_2_ respectively; shaking those mixtures with a speed of 150 rpm for 12 h at 15 °C, sitting them still for 2 h; and then, centrifuging for 10 min at 4000 rpm, filtering the supernatants with 0.45 μm millipore filter for further measuring. The same procedures were taken, but the experimental temperature was changed to 25 °C and 35 °C, respectively. The three temperatures set here was to check the effect of the temperature on adsorption.

The removal efficiency of the adsorbent was measured by Equation (1) and the adsorption capacity was calculated by Equation (2).

(1) $$r\;\left(\%\right)=\left(1-\frac{C_{1}}{C_{0}}\right)\times 100\%$$

where r (%) refers to the removal efficiency of the adsorbent, C_1_ (mg/L) refers to the final concentration of Cd(II) at the end of the adsorption and C_0_ (mg/L) refers to the initial Cd(II) concentration of the adsorbate solution.

(2) $$q=\frac{\left(C_{0}-C_{e}\right)}{m}\times V$$

where q (mg/g) refers to the adsorption capacity of the adsorbents, C_0_ (mg/L) refers to the initial concentration of the Cd(II) solution and C_e_ (mg/L) refers to the final concentration of Cd(II) at the end of the adsorption, V (L) is the volume of the Cd(II) solution, and m (g) is the mass of the added adsorbents.

### 2.6. Characterization Analysis of the Adsorbents

Five samples including four adsorbents and RWS were characterized by scanning electron microscope (SEM, JEOL, JEM-200CX, Kyoto, Japan) and Brunauer-Emmett-Teller surface area measurement (BET, BELSORP-miniII_Microtrac. BEL, Kyoto, Japan). The infrared spectra of RWS, ENMWS, EKMWS, EHMWS, and ECMWS were determined by Fourier transform infrared spectroscopy (FTIR, IR Affinity-1, Kyoto, Japan). For the measurement of SEM image, wheat straw powder was used and it was sprayed with gold for good electrical conductivity. For FTIR measurement, the operation steps were: mixing and grinding 1.0 mg WS sample with 100 mg potassium bromide (KBr, IR grade) to reduce light scatter, and then compressing to form a disk under 15 MPa for 2 min.

Cd(II) content was determined by atomic absorption spectroscopy (AAS, PEAA 700, Waltham, MA, USA). HF-HCl-HClO_4_-HNO_3_ digestion was conducted by a microwave digestion system (DS-360L, Guangzhou, China).

### 2.7. Statistical Analysis

Origin (8.0, OriginLab, Northampton, MA, USA), Excel (2007, Microsoft Corporation, Redmond, NM, USA), and SPSS (17.0, IBM, Armonk, NY, USA), were used for data analysis. The significant differences were detected by the least significant difference (LSD) test, regarding *p* < 0.05 as the statistically significant level. All the data quoted were mean values.

## 3. Results and Discussion

### 3.1. Removal of Cd(II) from Waterlogged Paddy Soil

#### 3.1.1. Effect of Different Pretreatment Methods on the Proportion Changes of Different Cd(II) Fractionations Remaining on the Soil

**Figure 1:**
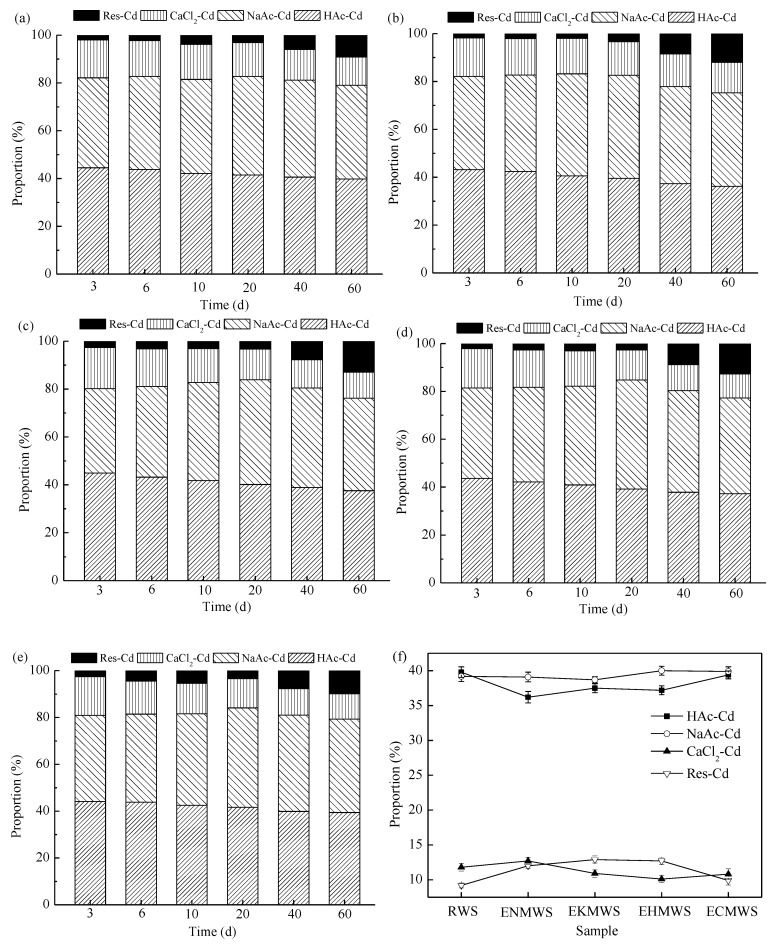
Proportions changes of different Cd(II) fractionations in soil as a function of time after adding different adsorbents. (**a**) Raw wheat straw (RWS) adsorbent; (**b**) ENMWS adsorbent; (**c**) EKMWS adsorbent; (**d**) EHMWS adsorbent; (**e**) ECMWS adsorbent; (**f**) Proportion changes of four Cd(II) fractionations among RWS, ENMWS, EKMWS, EHMWS, and ECMWS after 60-day incubation.

It was difficult to properly measure the amount of heavy metal ions in soil, because heavy metal ions usually presented different fractionations. Besides, the toxicity of heavy metals was not only concerned with the total amount, but was also decided by their fractionations to a large extent. According to this experiment, four Cd(II) fractionations were detected and the content change of each fractionation was compared after adding adsorbents. Figure 1 demonstrated the changes of the percentage contents of HAc-Cd, NaAc-Cd, CaCl_2_-Cd, and Res-Cd over time. The adsorption capacity of each adsorbent was different from each other, but in the soil, this difference was not as remarkable as in aqueous solution. The overall trend for the four adsorbents was that they showed higher Cd(II) adsorption capacity than that of the RWS when it came to the fractionations of HAc-Cd and CaCl_2_-Cd, and the adsorbent ENMWS had the highest Cd(II) removal efficiency among the four adsorbents. When HAc-Cd was chosen as an indicator to evaluate the removal rate, following result was found: by the end of the incubation, ENMWS, EKMWS, EHMWS, and ECMWS could remove 16.48%, 14.48%, 16.20%, and 10.66% of the HAc-Cd in the soil sample, while Cd(II) removal efficiency of RWS was 10.56%.

As shown in Figure 1, at the early incubation stage, HAc-Cd accounted for approximately 45% of all the Cd(II) contents in all five treatments. This part of Cd(II) was similar to acid dissolvable Cd(II) of the BCR sequential extraction procedure for HAc was used as extracting agent, and the proportion of HAc-Cd dropped during the incubation. The proportion of NaAc-Cd made up around one-third of the whole Cd(II) contents at the beginning, and it had a moderate rise in the first 20 days of the incubation, and then slowly decreased to a percentage that was lower than that of its initial proportion. The CaCl_2_-Cd could be deemed as a combination of water-soluble and part of exchangeable forms of Cd(II). According to the figures, the initial ratios of CaCl_2_-Cd were 15% or so in all samples, and with the passing of time it decreased slowly. At the end of the incubation, this proportion was decreased by 25–40%. This indicated that the mobility and bioavailability of Cd(II) changed after adding all five types of WS into the soil, and it also suggested that the adsorbents had a positive effect on reducing the toxicity of Cd(II) in soil, for the water-soluble fraction of Cd(II) could reflect the mobility of Cd(II) [7]. This phenomenon was in accordance with some of the previous researches [25,26,27]. As to the exchangeable Cd(II), Smolders et al. [28] thought that because of soil aging, it dropped slowly over time; while some believed that it was the increase in pH value that decreased the amount of exchangeable Cd(II) [29]. With the increase of soil pH, those organic matters in soil would be more easily to absorb Cd(II), which could be another reason for the increase of the water-soluble Cd(II). Because the overall variation trend of pH was very similar to the variation pattern of DOC content, it showed that pH and DOC content had close correlation with each other and they directly influenced the adsorption capacity of the adsorbents on Cd(II). With respect to the residual Cd(II), it increased along with incubation time, for the proportions of other fractionations of Cd(II) were more or less decreased and the total amount of Cd(II) was not changed.

#### 3.1.2. Changes of Soil pH and DOC Content during the Incubation

**Figure 2:**
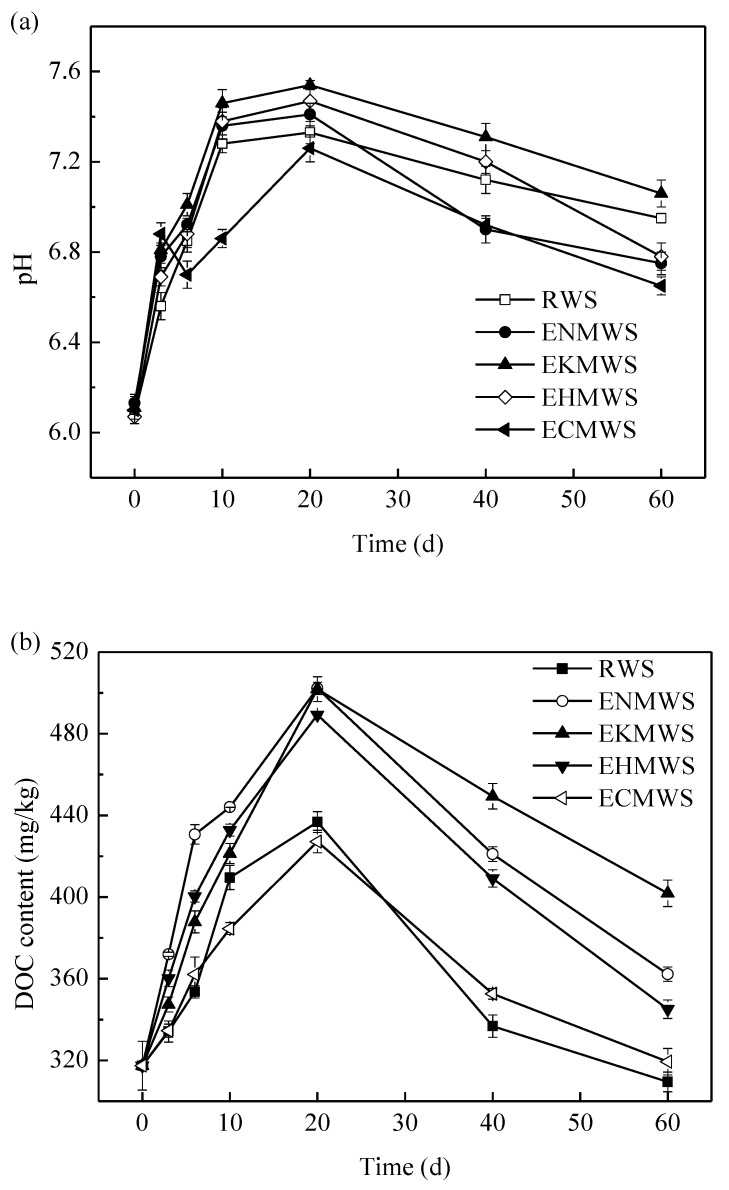
(**a**) Variation of soil pH over time after adding different adsorbents; (**b**) Variation of soil dissolved organic carbon (DOC) content over time after adding different adsorbents.

From Figure 2a, it could be seen that once the soil was mixed with WS and waterlogged, its pH gained a rapid rise in the first 10 days and peaked on the 20th day, and then started to drop slowly. From day 40–60, the pH value of the samples decreased slowly and then seemed to reach a relatively stable value at the end of the incubation, but it was still higher than the initial value of the samples. What lay behind the pH changes of soil samples might be that WS was hydrolyzed when it was mixed with the soils, and organic matters in the WS would be decomposed by microorganisms in the soil and those decomposition products might neutralize the acid in the soil samples [30,31]. When the WS was decomposed, the decarboxylation of organic anions of WS caused soil pH to increase, and the association/dissociation of organic compounds could cause the pH of the soil to rise and drop, depending on the initial pH of the soils [32]. In the first 20 days, those microorganisms in the soil decomposed the organic matters to a series of intermediate products that contain organic acids, which would increase the pH value of the soil [33]. Besides, due to the respiration of the microorganisms, the produced carbon dioxide dissolved in the water and formed CO_3_^2−^ and HCO_3_^−^, which could also increase the soil pH [34]. As for the decrease of pH in the late incubation stage, it might be that the ion Cd^2+^ bound the carbonate and produced CdCO_3_ [35]. Because CdSO_4_ was added to contaminate the soil samples, a part of SO_4_^2−^ might reduce to S^2−^, which would also combine with Cd^2+^ and therefore decrease the pH of the soil [36]. This result was a little bit controversial in different researches. For example, the study of Lourençon et al. [37] showed that hardwood and other softwood had the potential to increase soil pH due to their alkalinity. While Scharenbroch et al. [38] found that the soil pH had a tendency to decrease in the absence of WS. A blank control group where soil was incubated alone without adding any kind of WS, and the pH measurement results showed that it had no obvious change over time.

The changes of DOC during the incubation were presented in Figure 2b. The overall tendency of DOC changes was similar to the soil pH changes, except the control group (soil sample added with RWS), namely the sample that was mixed with RWS. In the control group, the DOC concentrations decreased over time, but changes were not as dramatic as the experimental groups (soil samples added with four kinds of pretreated WS, i.e., ENMWS, EKMWS, EHMWS, and ECMWS). The result was in consistent with the findings of Wang and Chen [39]. From Figure 2b, rapid increases could be seen in the first 20 days, which was probably because the addition of WS had greatly increased the total DOC content. The carbon content of WS usually was higher than 40%, its main components were cellulose, hemicelluloses and lignin [40]. When WS was returned to soil and put under waterlogged condition, it would decompose to a lot of intermediates (mainly organic acids with small molecular) with which were an important source of DOC [41,42]. Therefore, the DOC content of the samples increased during the earlier stage of incubation. Soil microorganisms decomposed the organic matters that come from WS and then new DOC was created, which would be served as carbon sources and energy for them to grow and reproduce. At the same time, the process of metabolism of microorganisms would increase the DOC content. Khodse and Bhosle [43] held the view that at the beginning of the decomposition, as the main composition of the dissolved organic matters were monosaccharide, amino acids, amino sugar, and so forth, microorganisms in the soil would proliferate rapidly. The phenomenon that the DOC contents got a sharp decline after 20 days proved that the microorganisms consumed a lot of organic matters. With the enhancement of the soil microbes’ activity, they started to decompose the cellulose and lignin of WS, together with the death residues of those microorganisms, the metabolic products would offset the DOC they consumed. Thus, the DOC concentration decreased slowly in the late incubation period. The final DOC concentrations of the samples increased but they still remained close to the initial ones, suggesting that a decomposition saturation stage of the microorganisms in the soil might be reached. About this phenomenon, previous literatures have explained that approximately 30–40% crop residues would be decomposed in the earlier period of incubation and further decomposition would be time-consuming and inefficient after burying in a field for 3–4 weeks [44,45].

Among all the adsorbents, ENMWS caused the greatest change of DOC content in the soil, followed by EKMWS, EHMWS, ECMWS, and RWS. A correlation test suggested that the changes of soil pH and DOC content had statistically positive correlation with each other (*p* < 0.05) but not rather significant, which was against some other researchers’ findings, such as Zhao et al. [46].

### 3.2. Removal of Cd(II) from Aqueous Solutions

#### 3.2.1. Effect of Different Pretreatment Methods on Removal Efficiency of Cd(II)

**Figure 3:**
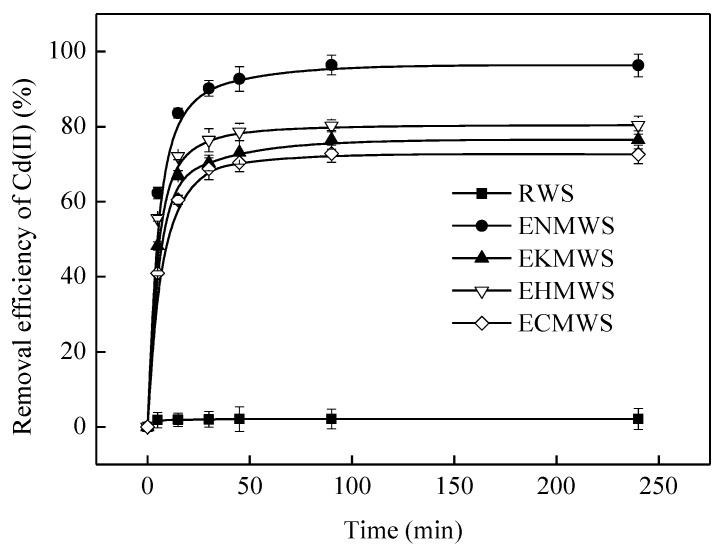
Removal efficiency of Cd(II) by RWS, ENMWS, EKMWS, EHMWS, and ECMWS (temperature of 25 °C, solid/liquid ratio of 10 g/L, solution concentration of 50 mg/L, initial pH value of 7.0).

Figure 3 described the removal efficiency of Cd(II) by different adsorbents as a function of the contact time. The removal efficiency of Cd(II) by the four adsorbents was much higher than the RWS, showing that the pretreatment methods improved the adsorption capacity of the WS. As suggested in Figure 3, the time period from 0 min to 90 min was when the major adsorption happened. The variation trend of all samples was similar to each other; the removal efficiency rose quickly in the first 45 min and reached to a stable value at the 90th min. In addition, the removal efficiency of the samples followed the order of ENMWS > EHMWS > EKMWS > ECMWS > RWS. Thus, the adsorbent ENMWS was the most effective one among all of the samples. This result suggested that 0.1 mol/L NaOH could change the structure of the WS more deeply than 0.1 mol/L KOH, for NaOH could damage the structural unit of the lignin more thoroughly, which was connected by ether linkage so that the lignin with high molecular weight would break down into smaller ones and be dissolved in solutions, so the WS might obtain more rough surface as well as larger specific surface area.

#### 3.2.2. Effect of Adsorbent Dosage on the Adsorption Capacity of the Pretreated WS

**Figure 4:**
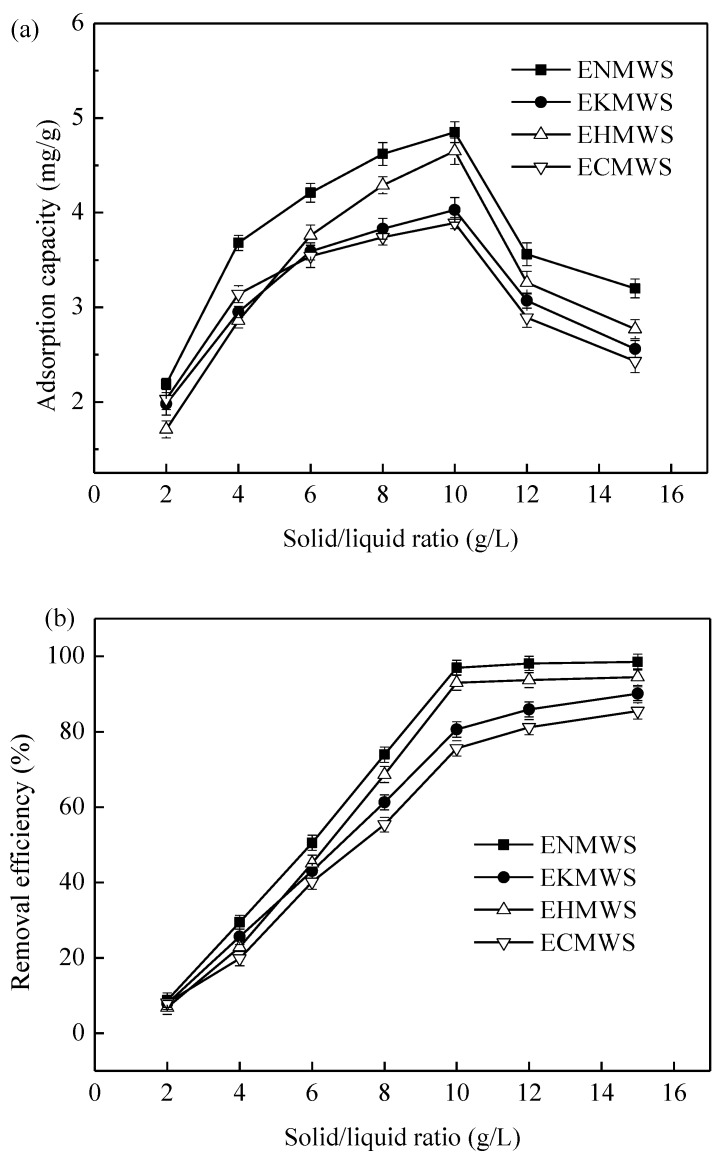
Effect of different solid/liquid ratio on the adsorption performance of the adsorbents (temperature of 25 °C, contact time of 90 min, solution concentration of 50 mg/L, initial pH value of 7.0). (**a**) Adsorption capacity of the adsorbents as a function of solid/liquid ratio; (**b**) Removal efficiency of Cd(II) by the adsorbents as a function of solid/liquid ratio.

Different dosages of the adsorbents added to the Cd(II) solution had a salient impact on the removal efficiency of the heavy metal ion. Seven solid/liquid ratios (solid/liquid ratio (g/L) here refers to the ratio of the mass of adsorbent (pretreated WS) and the volume of Cd(NO_3_)_2_ solution) were set to determine the optimum dosage of the adsorbents, and the result was presented in Figure 4a,b. Figure 4a showed the adsorption capacity of four adsorbents changed with the solid/liquid ratio. It could be seen that, as the solid/liquid ratio increased from 2 to 10, the adsorption capacity of the four adsorbents increased rapidly but the increment speed slowed down, and when the solid/liquid ratio continued to increase, the adsorption capacity decreased quickly. The highest adsorption capacity of all adsorbents was obtained at the solid/liquid ratio of 10 g/L, and the adsorbent ENMWS ranked the most effective among the four samples with the adsorption capacity of 4.85 mg/g. At the solid/liquid ratio of 10 g/L, the highest adsorption capacity of each adsorbent followed the series ENMWS > EHMWS > EKMWS > ECMWS > RWS, which also proved that ENMWS was the most effective adsorbent among the four. Figure 4b showed the relations between removal efficiency (calculated by Equation (1)) of Cd(II) and solid/liquid ratio. The overall trend for the removal efficiency of Cd(II) by four adsorbents was increased with the increase of the solid/liquid ratio, but when the solid/liquid ratio reached 10 g/L, the removal efficiency by ENMWS and EHMWS did not increase any more, while the EKMWS and ECMWS continued to increase. The reason was that the amount of Cd(II) in the aqueous solution was almost adsorbed by ENMWS and EHMWS, adding more adsorbents into the solution would not increase the removal efficiency remarkably. As EKMWS and ECMWS only had a removal efficiency of 75–80% when the solid/liquid ratio was 10 g/L, adding more adsorbents could increase the removal efficiency. When comparing Figure 4a,b, a difference was found between the variation pattern of adsorption capacity and removal efficiency. A possible explanation was that a higher solid/liquid ratio meant that more adsorbent was added when the total volume of the Cd(II) solution remained the same. This suggested that the amount of active sites located on the surface that could be used to adsorb Cd(II) increased, making the removal efficiency of Cd(II) increased. Meanwhile, when the adsorbent dosage increased more adsorption sites might not be used sufficiently as the total Cd(II) amount did not change. Therefore, the adsorption capacity of the adsorbents dropped down when the solid/liquid ratio was higher than 10 g/L. When considering both adsorbent dosage and removal efficiency of Cd(II), the optimum solid/liquid ratio was 10 g/L in this experiment.

#### 3.2.3. Effect of pH on Cd(II) adsorption

**Figure 5:**
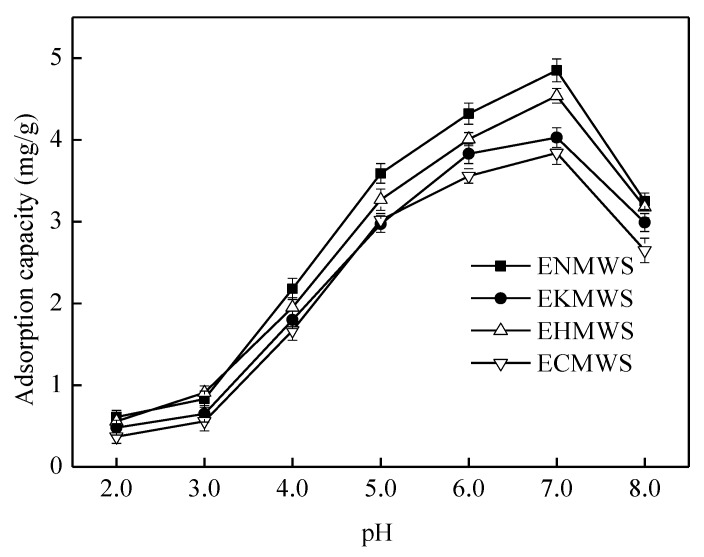
Effect of initial pH of the Cd(II) solution in the adsorption capacity of the adsorbents (solid/liquid ratio of 10 g/L, 25 °C, contact time of 90 min, solution concentration of 50 mg/L).

Figure 5 showed the adsorption capacity of Cd(II) by four adsorbents when the initial pH of the solution increased from 2.0 to 8.0. The adsorption capacity rose with the pH value of the Cd(II) solution, and the highest adsorption capacity of ENMWS, EKMWS, EHMWS, and ECMWS was 4.85, 4.03, 4.54, and 3.84 mg/g, respectively at the pH of 7.0. An almost linear adsorption performance was observed for all adsorbents when the pH value increased from 3.0 to 5.0. In the pH variation section of 5.0 to 7.0, the increment speed slowed down and the adsorption capacity was increased by 35–38%. When the pH value was higher than 7.0, the adsorption capacity of the adsorbents dropped down rapidly, because the precipitate of Cd(II) and hydroxyl radicals would be formed under alkaline condition, which impeded the adsorption process [47]. Therefore, the suitable initial pH of the Cd(II) solution was 7.0 in the experiment, which was favorable to the adsorption process. A possible explanation was that, at low pH, Cd(II) suffered competitive adsorption with hydrogen ions, which decreased the removal efficiency of Cd(II) [48]. As the pH of the solution increased, the competitive adsorption effect of hydrogen ions weakened while the amount of negative charges existed on the surface of the adsorbents increased, which would offset some of the electrostatic repulsion between adsorbents and the Cd(II) [49]. For all these reasons, the adsorption performance of Cd(II) by the adsorbents improved obviously. The composition of the WS was complicated and it mainly included cellulose, hemicellulose, lignin, soluble sugars, and so on. These compositions had many surface active groups, such as hydroxyl and carboxyl, which could combine with heavy metal ions by hydrogen bonding or ions exchange [50]. Possible mechanism for the combination of Cd(II) and the adsorbents were listed, as follows.

2(-ROH) + Cd^2+^ → (RO)_2_Cd + 2H^+^ (ion exchange)

2(-RCOOH) + Cd^2+^ → (RCOO)_2_Cd + 2H^+^ (ion exchange)

2(-ROH) + Cd(OH)_2_ → (-ROH)_2_Cd(OH)_2_ (H-bonding)

2(-RCOOH) + Cd(OH)_2_ → (-RCOOH)_2_Cd(OH)_2_ (H-bonding)

#### 3.2.4. Adsorption Kinetics Analysis

**Figure 6:**
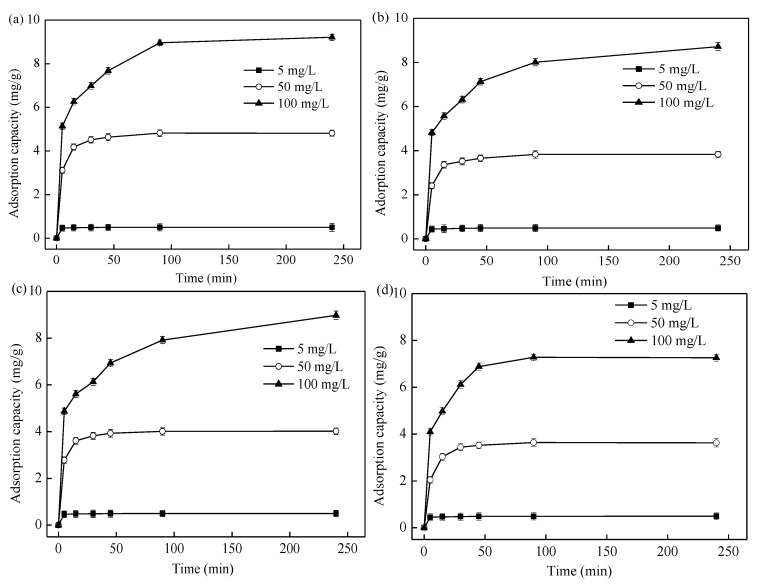
Adsorption performance of the four kinds of adsorbents as a function of contact time (solid/liquid ratio of 10 g/L, temperature of 25 °C, initial pH value of 7.0). (**a**) ENMWS; (**b**) EKMWS; (**c**) EHMWS; and, (**d**) ECMWS.

Figure 6 showed the adsorption performances of ENMWS, EKMWS, EHMWS, and ECMWS as a function of contact time. Three initial concentrations: 5, 50, and 100 mg/L were set to fit the adsorption curves. The adsorption process was very fast for all the adsorbents, and it took 90 min to reach the adsorption equilibrium for the adsorption capacity did not have any obvious change. The first 15 min for the adsorption process was a fast adsorption, and a slow one appeared from 15 min to 90 min. The intermolecular interaction forces were mainly the Van der Waals force and hydrogen bond force at the fast adsorption stage, and they happened within a short time [51]. In the slow adsorption phase, pore diffusion was the major process. When the initial Cd(II) concentration was 5 mg/L, the adsorption capacity of all adsorbents was rather low, but the removal efficiency was high at the beginning of the adsorption. This was because the amount of Cd(II) was certain, and so adding more adsorbents into the Cd(II) solution would speed up the removal rate of the adsorbents. When the initial Cd(II) concentration got higher, the same amount of adsorption sites were occupied by some Cd(II) quickly, leaving the rest of Cd(II) waiting to be absorbed by the soil, which took longer time to peak the maximum adsorption quantity.

Three sorption kinetic models based on reaction order were employed to fitting the adsorption of Cd(II) onto the adsorbents: the pseudo-first-order model (PFOM), the pseudo-second-order model (PSOM), and Weber and Morris intra-paticle diffusion model (WMM). The equations of the three models were given by Equations (3)–(5), respectively.

(3) $$\log\left(q_{1}-q_{t}\right)={\mathrm{logq}}_{1}-\frac{k_{1}}{2.303}t$$

(4) $$\frac{t}{q_{t}}=\frac{1}{k_{2}q_{2}^{2}}+\frac{1}{q_{2}}t$$

(5) $$q_{t}=k_{p}t^{\frac{1}{2}}+C$$

where k_1_ (min^−1^), k_2_ (g/(mg·min)), and k_p_ (g/(mg·min^1/2^)) are the first order rate constant, second order rate constant, and Weber and Morris intra-particle diffusion rate constant, respectively; q_t_ (mg/g) is the adsorption amount at time t (min), q_1_ (mg/g) and q_2_ (mg/g) are the calculated values of the adsorption according to the models; and, C (mg/g) refers to the thickness of the boundary layer.

As regards the solving of the equations, linear fitting method was taken. For example, k_1_ could be determined by the slope of liner plots of log(q_1_ − q_t_) versus t while q_1_ was calculated by the intercept (with a assumptive value at the beginning). The same pattern could be followed to determine k_2_, q_2_, and k_p_, C.

The correlation coefficients (R^2^) value of the PFOM, PSOM, and WMM was about 0.6, 0.9, and 0.7 respectively, showing that the PSOM was the best fitted for Cd(II) adsorption onto the adsorbents, and the kinetic parameters of the PSOM are presented in Table 1. It could be seen that the R^2^ value of the PSOM for all samples were higher than 0.99 and the calculated adsorption capacity value q_2_ was also close to the experimental value q_e_. These results were in accordance with most kinetic studies that using straw biosorbents to adsorb heavy metal ions from aqueous solutions [52,53].

As the PSOM is based on the assumption that the adsorption rate is limited by the chemical sorption mechanism between adsorbents and adsorbates, including the sharing and transfer of electron pair bond, the rate-limiting step of adsorption might be chemical sorption or chemisorption involving valency forces [54]. It was appropriate to predict that the adsorption behavior might involve valency forces through sharing or exchanging electrons between transition cadmium ions and adsorbents. Many other studies on the adsorption kinetics of Cd(II) on various adsorbents have also reported the same results [55,56].

#### 3.2.5. Adsorption Isotherms Analysis

Adsorption isotherms are significant to investigate the relationship between metal ions bond and biosorbent materials. Data of adsorption of Cd(II) onto the adsorbents were analyzed with three common adsorption isotherm models in which Langmuir, Freundlich, and Temkin models were included. Langmuir isotherm model is based on the assumption that a monomolecular layer is formed when adsorption starts, and no other interaction between the adsorbed molecules will happen [57,58]. The Freundlich isotherm model can be applied to multilayer adsorption, with non-uniform distribution of adsorption heat and affinities over the heterogeneous surface. As for the Temkin isotherm model, it contains a factor that explicitly takes into account adsorbent-adsorbate interactions [59]. Equations (6)–(8) could express those three models, respectively.

(6) $$\frac{1}{q_{s}}=\frac{1}{K_{L}Q_{m}c_{e}}+\frac{1}{Q_{m}}$$

(7) $${\mathrm{lgq}}_{s}={\mathrm{lgK}}_{F}+\frac{1}{n}{\mathrm{lgc}}_{e}$$

(8) $$q_{s}=\frac{\mathrm{RT}}{\Delta Q}{\mathrm{lnK}}_{T}c_{e}$$

where q_s_ (mg/g) is the amount of Cd(II) adsorbed of by the adsorbents; c_e_ (mg/L) is the concentration of Cd(II) in simulated solution; K_L_ (L/mg), K_F_ (L/g), and K_T_ (L/g) are the Langmuir constant, Freundlich constant, and Temkin constant, respectively; Q_m_ (mg/g) describes the maximum adsorption capacity of the adsorbents; n is also a Freundlich constant that relates to the adsorption capacity and adsorption intensity; R is the universal gas constant whose value is 8.314 J/(K·mol); ΔQ (kJ/mol) is the variation of the adsorption energy; and, T (K) refers to the temperature in Kelvin.

Adsorption isotherm parameters of the Langmuir, Freundlich, and Temkin models were presented in Table 2. The three temperatures, i.e., 288, 298, and 308 K recorded in Table 2 were Kelvin temperature, which was the same as 15, 25, and 35 °C. When comparing these fitting data, it could be found that both the Langmuir and Temkin isotherm model had good correlation with the adsorption process, and the Langmuir isotherm model was the fittest one for its correlation coefficient R^2^ at three temperatures were higher than 0.99. The R^2^ value of Freundlich isotherm model was about 0.92–0.96, showing that this model was not suitable to describe the adsorption process of the experiment. In addition, according to Table 2, the maximum adsorption capacities of the adsorbents calculated by Langmuir isotherm model were very close to the experimental ones. Accordingly, the adsorption process could be well described by the Langmuir model, which indicated that the adsorption was a monolayer adsorption, and the adsorption was mainly occurred on the active surface of the adsorbents [60,61]. The amount of Cd(II) adsorbed by the adsorbents was the summation of adsorption on all adsorption sites (each has bond energy), with the stronger binding sites being occupied first, until adsorption energies were exponentially decreased upon the completion of the adsorption process [62]. Besides, the maximum adsorption capacity Q_m_ of the adsorbents increased with the increase of temperature, suggesting that Cd(II) had higher affinity with the adsorbents when the temperature was high. This might also suggest that the adsorption involved in this experiment was a combination of physical adsorption and chemical adsorption process for temperature was a factor that could influence the adsorption [63,64].

### 3.3. Characterization Analysis

**Figure 7:**
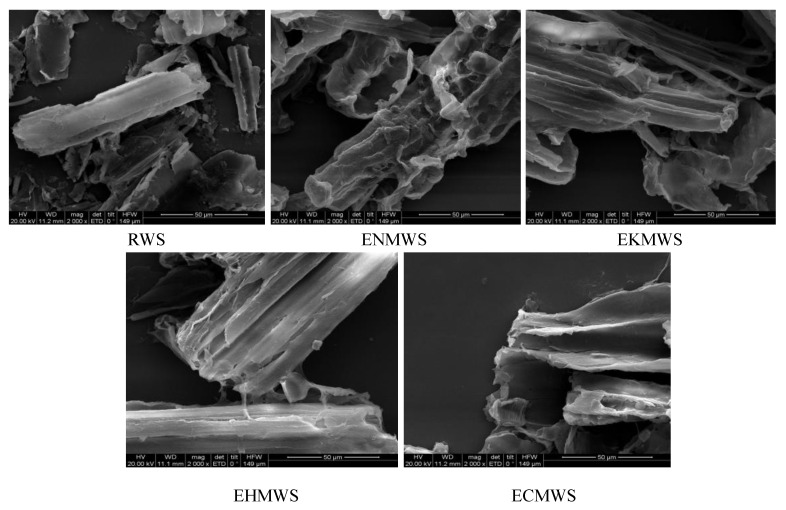
Scanning electron microscope (SEM) images of RWS, ENMWS, EKMWS, EHMWS, and ECMWS.

The SEM images for RWS, ENMWS, EKMWS, EHMWS, and ECMWS were shown in Figure 7, and the scale was 100 μm. From the image of RWS, a tube-like structure with a relatively dense and continuous surface could be seen. SEM images of all the adsorbents showed rugged and coarse surfaces when comparing with the RWS, their structures seemed to be torn and broken, besides, split and fibrillation could be observed. The SEM results were in keeping with specific surface area analysis that the BET data for RWS, ENMWS, EKMWS, EHMWS, and ECMWS were 0.616, 4.256, 3.132, 3.985, and 2.073 m^2^/g, respectively. It clearly showed that the pretreated WS had larger specific surface area than the RWS, and it could reflect the adsorption capacity of the adsorbents indirectly, for larger specific surface area meant higher adsorption capacity.

**Figure 8:**
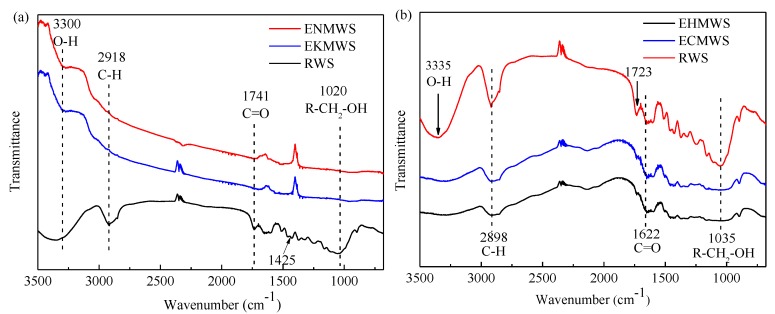
Fourier transform infrared spectroscopy (FTIR) spectra of the RWS and pretreated WS: (**a**) RWS, ENMWS, and EKMWS; (**b**) RWS, EHMWS, and ECMWS.

The FTIR spectra were presented in Figure 8. From the table, it could be seen that the characteristic peaks of the RWS were located at the wavenumber of 3750–3000 cm^−1^ (O–H stretching vibration region), 3000–2800 cm^−1^ (saturated C–H stretching vibration region, such as -CH_3_ and -CH_2_), 2400–2100 cm^−1^ (triple bond and cumulative double bond stretching vibration region), 1900–1630 cm^−1^ (C=O stretching vibration region), 1475–1300 cm^−1^ (saturated -CH in-plane bending vibration region), and 1065–1015 cm^−1^ (R-CH_2_-OH stretching vibration region). Differences could be observed when comparing the FTIR spectra of those pretreated WS with the RWS. The ENMWS and EKMWS shared similar characteristic peaks and functional groups, such as -CH and -COO, disappeared. The reason might be that the alkaline pretreatment could cause degradation of cellular compounds, which included proteins, cell wall, and some complex organic components of the biomass. For the EHMWS and ECMWS, the missing functional groups were C=C and -COO, besides the amide characteristic peak region (1690–1630 cm^−1^), the C=O was also found in acid region (1725–1700 cm^−1^). The result was in line with some previous studies that showed that acid oxidation could introduce new functional groups to the biomass, and sometimes it could also change the original functional groups of the biomass [65].

## 4. Conclusions

WS pretreated by ethanol and microwave-assisted alkalization or acid oxidation obtained higher adsorption capacity and removal efficiency of Cd(II) from aqueous solutions. The highest removal efficiency of Cd(II) was 96.4% and the maximum adsorption capacity was 4.82 mg/g achieved by the adsorbent ENMWS with initial Cd(II) solution concentration of 50 mg/L, the solid/liquid ratio of 10 g/L, pH of 7.0, and temperature of 25 °C. Among Langmuir, Freundlich, and Temkin isotherm models, the Langmuir model best fit the adsorption process of Cd(II). The adsorption of Cd(II) by all adsorbents could be described better by the PSOM than by the PFOM or WMM, which indicated that it was a chemical process. For the removal of Cd(II) from soil, the proportion of HAc-Cd and CaCl_2_-Cd decreased with the incubation time, suggesting that the toxicity of Cd(II) in soil reduced. Both pH value and DOC content of the soil sample changed obviously during the incubation, and a positive correlation was found in both variables (*p* < 0.05). Characteristic analysis proved that the pretreatment methods changed the structure of the WS for rougher surface and larger specific surface area was observed. FTIR results showed that some functional groups of the WS were changed after the pretreatment.

## Figures and Tables

**Table 1:** Kinetic parameters of the pseudo-second-order model for Cd(II) adsorption onto four kinds of adsorbent.

Adsorbent	C_0_ (mg/L)	q_e_ (mg/g)	q_2_ (mg/g)	k_2_ (g·mg^−1^·min^−1^)	R^2^
ENMWS	5	0.498	0.499	4.52	1.0000
	50	4.815	4.871	0.09	0.9999
	100	9.211	9.515	0.01	0.9991
EKMWS	5	0.500	0.502	1.59	1.0000
	50	3.830	3.877	0.10	0.9999
	100	8.720	9.025	0.01	0.9988
EHMWS	5	0.497	0.498	3.68	1.0000
	50	4.020	4.055	0.14	1.0000
	100	8.980	9.328	0.01	0.9972
ECMWS	5	0.497	0.499	2.39	1.0000
	50	3.630	3.681	0.10	0.9998
	100	7.260	7.440	0.03	0.9995

**Table 2:** Isotherm parameters for Cd(II) adsorbed by ENMWS, EKMWS, EHMWS, and ECMWS.

Adsorbent	Temperature (K)	Langmuir			Freundlich			Temkin		
		Q_m_ (mg/g)	K_L_ (L/mg)	R^2^	n	K_F_ (L/g)	R^2^	ΔQ (kJ/mol)	K_T_	R^2^
ENMWS	288	11.51	0.0489	0.9912	1.8650	1.2148	0.9563	824.70	0.3192	0.9948
	298	12.32	0.0357	0.9966	2.1281	1.0510	0.9435	991.70	0.4244	0.9957
	308	14.27	0.0258	0.9932	2.4582	1.4048	0.9301	1128.96	0.6019	0.9899
EKMWS	288	10.25	0.0514	0.9967	1.9810	1.1071	0.9505	904.72	0.3621	0.9961
	298	11.18	0.0386	0.9958	2.1973	1.0442	0.9405	1091.73	0.4569	0.9949
	308	12.97	0.0301	0.9974	2.5240	1.3219	0.9278	1282.92	0.6446	0.9882
EHMWS	288	11.01	0.0501	0.9989	1.9604	1.0681	0.9515	845.97	0.3542	0.9960
	298	12.15	0.0368	0.9918	2.1529	1.0732	0.9425	1002.90	0.4356	0.9954
	308	13.89	0.0293	0.9926	2.4894	1.3804	0.9290	1186.45	0.6221	0.9891
ECMWS	288	9.76	0.0607	0.9986	2.2051	1.2980	0.9402	984.47	0.4607	0.9948
	298	10.34	0.0412	0.9984	2.2712	1.0421	0.9374	1196.72	0.4941	0.9938
	308	11.99	0.0389	0.9965	2.7337	1.5014	0.9208	1371.05	0.8028	0.9825
